# Optimally Controlled Diabetes and Its Influence on Neonatal Outcomes at a Level II Center: A Study on Infants Born to Diabetic Mothers

**DOI:** 10.3390/medicina59101768

**Published:** 2023-10-04

**Authors:** Mihai Muntean, Irina Prelipcean, Maria-Andreea Racean, Manuela Cucerea, Amalia Fagarasan, Carmen Tamara David, Claudiu Marginean, Laura Mihaela Suciu

**Affiliations:** 1 Department of Obstetrics and Gynecology, University of Medicine Pharmacy Science and Technology George Emil Palade of Târgu Mures, 540142 Târgu Mures, Romania; munteanmihai@yahoo.com (M.M.); marginean.claudiu@gmail.com (C.M.); 2 Department of Neonatology, University of Rochester Medical Center Golisano Children’s Hospital at Strong, Rochester, NY 14642, USA; 3 Department of Neonatology, University of Medicine Pharmacy Science and Technology George Emil Palade of Târgu Mures, 540142 Târgu Mures, Romania; marry_deea@yahoo.com (M.-A.R.); manuelacucerea@yahoo.com (M.C.); 4 Department of Pediatric Cardiology, University of Medicine Pharmacy Science and Technology George Emil Palade of Târgu Mures, 540142 Târgu Mures, Romania; amalia_fagarasan@yahoo.com; 5 Faculty of Medicine, University of Medicine Pharmacy Science and Technology George Emil Palade of Târgu Mures, 540142 Târgu Mures, Romania; carmentdavid@yahoo.com

**Keywords:** infants of diabetic mothers, optimally controlled diabetes, antenatal, neonatal outcomes

## Abstract

*Background and Objectives*: We investigated the effect of optimal maternal glycemic control on neonatal outcomes among infants born to mothers with diabetes. *Materials and Methods*: In this prospective study, we assessed 88 eligible mothers admitted to the obstetrics department for pregnancy evaluation. Our analysis included 46 infants born to diabetic mothers (IDMs) and 138 infants born to unaffected mothers, all admitted to the Level II Neonatal Intensive Care Unit (NICU). *Results*: Mothers affected by diabetes were generally older and exhibited a higher body mass index (BMI) and a greater number of gestations, although parity did not differ significantly. Cesarean section emerged as the most frequently chosen mode of delivery. A significantly higher proportion of infants in the affected group presented with respiratory disease (3% vs. 19.5%), which required NICU admission (4.3% vs. 23.9%), phototherapy (18.1% vs. 43.5%), and had congenital heart defects or myocardial hypertrophy (15.2% and 26% vs. 3% and 4.3%) compared to matched controls (*p* < 0.05). *Conclusions*: This study underscores the persistence of adverse neonatal outcomes in IDMs, even when maternal glycemic control is optimized. It calls for further investigation into potential interventions and strategies aimed at enhancing neonatal outcomes in this population.

## 1. Introduction

Diabetes in pregnancy is a significant health concern that can adversely affect maternal and fetal health [1]. Previous reports form the World Health Organization, the International Association of Diabetes and Pregnancy, and the American Diabetes Association have shown a 17% prevalence of hyperglycemia and a 6–9% prevalence of diabetes in pregnancy, with the majority (99%) being gestational diabetes [2].

Diabetes mellitus (DM) is a systemic chronic metabolic disorder characterized by increased insulin resistance and/or β-cell defects. It affects all ages from fetal life, newborn period, and childhood to late adulthood [3]. As obesity and diabetes prevalence have increased over the past several decades, more women are overweight and diabetic in the first trimester and many more pregnant women are diagnosed with gestational diabetes. 

IDMs are at an increased risk of adverse neonatal outcomes, including respiratory distress, growth restriction, hypoglycemia, and congenital malformations [4,5]. Since maternal health conditions mediate these risks, it is critically important to monitor maternal health both before and during pregnancy to anticipate the severity of neonatal adverse outcomes [4]. 

By screening all pregnant women for diabetes, good glycemic control and active management of their infants has been shown to reduce perinatal morbidity and mortality [5]. The prevention of gestational diabetes could be attained through pre-pregnancy prevention of obesity, weight management, increased physical activity, and good nutritional strategies [3]. If gestational diabetes cannot be prevented, strict control of the disease during pregnancy is necessary to avoid harming the future offspring. However, glycemic control during pregnancy can be challenging due to the maternal need for higher caloric intake and higher insulin resistance [6].

Randomized controlled trials demonstrate that the treatment of gestational diabetes reduces macrosomia, which is a significant risk factor for perinatal complications [7,8]. Besides optimal obstetric management, communication between obstetrics and neonatology is critical to plan for possible neonatal resuscitation, assessment, and care of IDMs [9]. To address the long-term complications, which carry the greatest public health significance, we need to deepen our understanding of how the intrauterine environment, shared genetic factors, and postnatal environment collectively contribute to these issues.

As a result of this study, we seek to further enhance our comprehension of the impact of optimal glycemic control on neonatal outcomes among IDMs. The main objective is to identify whether there is a comparable neonatal morbidity between infants of optimally controlled gestational and pregestational diabetic mothers and matched controls.

## 2. Materials and Methods

### 2.1. Study Setting and Sampling

A prospective observational case–control study was conducted at a Level II NICU with an annual birth rate of 1500 infants in the County Hospital Mures, Romania from January 2022 to March 2023, comparing IDMs with a control group of non-IDMs. The size of the affected group was chosen by convenience [38 cases with gestational diabetes (G-DM) and 8 cases with pregestational diabetes (PG-D) Type 1 Diabetes Mellitus (T1DM)]. We matched the affected group 1:3 for gestational age to a group of low-risk pregnancies at the time of inclusion in the study (138 low risk pregnant women). 

### 2.2. Study Protocol

Pregnant women with known pregestational T1DM and those diagnosed during their second to third trimester with gestational diabetes, matched for the gestational age (GA) healthy pregnant control group, were closely followed up during the third trimester of pregnancy as part of routine care. Mothers were followed in the outpatient obstetric department, which maintained a protocol with self-monitoring blood glucose, physical activity advice, and dietary intervention for mothers with gestational diabetes (a detailed lifestyle guidance was included in Supplementary Materials). Maternal diabetes mellitus was diagnosed according to the International Association of Diabetes and Pregnancy Study Group criteria, which are based on a 75 g oral glucose tolerance test at 24 to 28 weeks gestation [10]. 

Gestational diabetes (G-DM) was diagnosed by the presence of an elevated 1 h glucose tolerance test (serum glucose > 180 g/dL^−1^), or an elevated 2 h glucose tolerance test (serum glucose > 199 g/dL^−1^) in the absence of a diagnosis of diabetes before pregnancy in accordance with the International Classification of Diseases 10th [Revision], ICD-10 (O244). None of the G-DM mothers were on insulin treatment during the gestational and post-gestational period. 

Pregestational diabetes (PG-DM) or known preexisting diabetes was considered in the presence of plasma glucose level ≥125 mg L^−2^ or HbA1c of 5.8–6.4%, at some time during the five years before pregnancy in accordance with the ICD-10 (ICD-10: E10–E14, O240–O241). All mothers with PG-D exhibited Type 1 Diabetes Mellitus (T1DM), had a diabetes duration exceeding 5 years, and received subcutaneous insulin injections throughout both their gestational and post-gestational periods. 

Optimally controlled diabetes was defined by a fasting glucose level less than 95 mg dL^−1^ or a 2 h postprandial glucose level less than 120 mg dL^−1^ [11]. The self-measured fasting glucose level and the 2 h postprandial glucose level was recommended during the third trimester of pregnancy until spontaneous or induced birth after 37 weeks of gestation.

**Figure 1:**
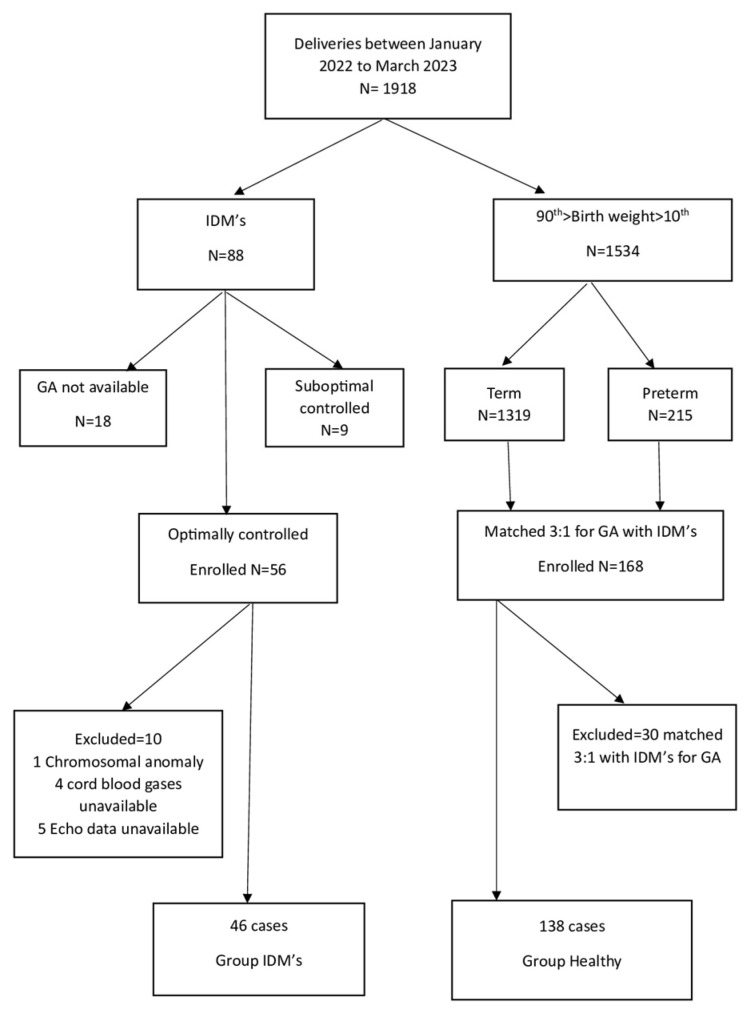
Flow chart of study participants.

Mothers with pregestational and gestational diabetes were advised to obtain a self-measured glucose level at least 10 times in a two-week interval. A fasting or a two-hour postprandial glucose level within the specified limits was considered as optimally controlled diabetes. If more than 3 out of 10 measurements were above target for a two-week period, the case was categorized as uncontrolled diabetes, treated by a multidisciplinary team, and excluded from the study. All included pregnant women were seen every two weeks or sooner, as clinically indicated. In between visits, the obstetric team could be contacted if necessary. Women were divided into pregestational diabetes (PG-D) and gestational diabetes (G-DM) groups. Among the affected mothers, G-DM was diagnosed at 28 weeks of gestation. Another 8 women with Type I preexisting diabetes were prospectively followed up closely until delivery. All recruited diabetic pregnant women received lifestyle guidance with diet, physical activity, and weight loss recommendations (see Supplementary Materials). This management reflects the therapeutic practices seen in this region. Optimally controlled G-DM was achieved during the second to third trimester of pregnancy in 80% (56 of 70 recruited mothers). A 3-fold larger group of healthy, low risk pregnant women was used to perform comparative analyses regarding perinatal and neonatal outcomes (Figure 1).

Mothers were approached again at term (>37 weeks of gestation) and asked to participate in the study. The delivery team and the attending neonatologist were blinded to the subject’s participation into the study; data abstractors were not blinded to group allocation. Parental informed consent was obtained before active labor; those who agreed to participate and signed the consent form were included. Relevant prenatal and postnatal information were retrieved from the medical record. 

Ethical approval was obtained from the University of Medicine Pharmacy Science and Technology George Emil Palade of Târgu Mures, Romania, Ethics Review Board (No. 9143/05/2021).

Eligibility criteria:

IDMs cases were included if they satisfied the following eligibility criteria:

1. Infants born after 37 completed weeks of gestation
2. Mothers with optimally controlled gestational and pregestational diabetes
3. Singleton pregnancies with documented gestational age based on first trimester ultrasound and closely followed up until spontaneous or induced labor and delivery

Non-IDM controls were included if they were from low-risk singleton pregnancies with documented normal oral glucose tolerance tests and gestational age assessed based on first trimester ultrasound exam available. 

Exclusion criteria were as follows:

4. Maternal age < 18 years
5. Mothers with uncertain etiology for the diagnosis of diabetes (ICD-10: E12–E14)
6. In utero fetal deaths and medical termination of pregnancy
7. Pregnancies of unknown gestational age
8. Pregnancies with fetal abnormalities, preeclampsia and eclampsia (ICD-10: O14), and gestational hypertension (ICD-10: O10)
9. Suboptimal controlled gestational and pregestational diabetes
10. Infants born at less than 37 weeks or after 42 weeks of gestational age
11. Multiple pregnancies
12. Infants with genetic syndromes or chromosomal anomalies

Maternal data included maternal age, anthropometric data recorded at the time of delivery, antenatal history, and family history of diabetes, mode of delivery. Neonatal data included gestational age at delivery, birth weight, cranial perimeter and height, APGAR score at 1- and 5 min after birth, and arterial cord blood pH, which is standard of care in our department and was performed with an I-STAT Analyzer (MN:300-G, Abbot Point of Care Inc. N.J., USA). After birth, the clinical status of the newborns was evaluated, and the NICU admission was performed at the discretion of the attending neonatologist. 

Neonatal complications were defined as follows:

13. Hypoglycemia = blood glucose < 35 mg dL^−1^
14. Respiratory disorder = any form of positive pressure for optimal oxygenation
15. Polycythemia = hematocrit >60%
16. Phototherapy = intervention for the treatment and prevention of severe hyperbilirubinemia. The threshold for phototherapy was calculated on bilirubin nomogram
17. Hyperbilirubinemia = levels requiring phototherapy according to the clinical practice for chronological age [12]
18. Myocardial hypertrophy = interventricular septum thickness >5 mm on echocardiography (Q218)
19. Congenital heart defects were classified according to ICD-10 definitions (Q-210 Ventricular septal defect, Q-211 Atrial septal defect, Q-208 Other congenital malformations of cardiac chambers and connections, Q-213 Tetralogy of Fallot, Q-249 Congenital malformation of heart, unspecified, Q-250 Patent ductus arteriosus)

### 2.3. Statistical Analysis

Continuous variables were reported as mean and standard deviation, or as median and range, and categorical variables were reported as numbers and percentages. An independent sample T test was used to assess the differences between groups. Logistic regression tests included Lemeshow and Hosmer, −2Log likelihood, Cox and Snell R Square, and Nagelkerke R Square test. Multivariate logistic regression models were used to examine the association between antenatal data and the outcomes. A *p* value < 0.05 was considered statistically significant. Analyses were performed in SPSS (Version 21.0, Chicago, IL, USA). 

## 3. Results

The flowchart of the study population is shown in Figure 1.

### 3.1. Study Population and Maternal Characteristics

Univariate comparison revealed that, overall, diabetic mothers were older and had higher BMI based on weight than unaffected mothers. Among diabetic mothers, those with G-DM had a higher weight at the time of delivery compared to those with PG-DM and healthy mothers (Table 1). Family history of diabetes was higher among those with PG-DM, but the difference was not statistically significant (*p* > 0.05). The number of gestations was higher among affected mothers, but the parity was not different between groups. The Cesarean section was more often the mode of delivery among affected mothers, and none of the mothers delivered vaginally among PG cases. Meconium-stained amniotic fluid and blood cord pH were not different between the groups, however, the need for oxygen use in the delivery room was higher among both affected groups, and Apgar score at 1- and 5 min after birth was lower among IDMs (*p* < 0.05). 

### 3.2. Neonatal Characteristics

Of the 46 IDMs, gestational age at birth was lower among mothers detected pre-pregnancy with diabetes compared to diabetic mothers detected during the second to third trimester of pregnancy (*p* < 0.01). The mean birth weight and length was higher among IDMs compared to healthier controls (*p* < 0.05). More often, G-IDM were on higher percentiles for weight and on lower percentiles for length on Fenton’s growth charts compared to PG-IDM and non-affected groups, and no differences of mean cranial perimeter and percentiles were found between groups (Table 2).

### 3.3. Neonatal Outcomes

There was a significantly higher proportion of infants in the affected group who had respiratory disease (3% vs. 19.5%), needed admission to the NICU (4.3% vs. 23.9%), and required photography (18.1% vs. 43.5%) compared to the control group (*p* < 0.05). Congenital heart defects and myocardial hypertrophy was detected more often among the affected group (15.2% and 26% vs. 3% and 4.3%) (*p* < 0.05), but no significant differences were detected between PG-DM and G-DM infants (*p* > 0.05). The mean length of hospital stay was longer among the affected group (4.7 ± 2.7 vs. 3.5 ± 1.3 days), with the longest being in the PG-DM group (*p* < 0.05) (Table 3).

In terms of the most severe outcomes, the unadjusted comparison revealed significantly higher odds of respiratory disease, congenital heart defects, and the need for NICU admission among IDMs compared to infants of non-affected mothers (Table 4). 

We ran a binary logistic regression model, with respiratory disease being the dependent variable and PG-DM, G-DM, male sex, mode of delivery and familial history of diabetes as covariates. A good model of fit was evident by a non-significant Hosmer–Lemeshow test (*p* = 0.56). The analysis revealed higher impact of PG [OR 8.9 (95% CI: 1.3–61.2)] and G-DM [OR 6.5 (95% CI: 1.8–24.1)], but not a significant impact for the mode of delivery [OR 3.1(95% CI: 0.7–13.7)], male sex [OR 0.9 (95% CI: 0.3–3.2)] or familial history of diabetes [OR 1.4 (95% CI: 0.3–6.4)] on the neonatal respiratory outcome. 

## 4. Discussion

The primary objective of this study was to estimate the neonatal outcomes of infants of optimally controlled diabetic mothers compared to infants of low-risk pregnant women admitted to one academic Level II Neonatal Intensive Care Center. We found that in singleton pregnancies, the optimally controlled glycemic level during the last weeks of pregnancy was not associated with a reduction of the maternal and neonatal complications. Current results showed that among the G-DM population, the mean of maternal age, weight, BMI and gestation rank tended to be higher. This is consistent with studies of Galtier and Wendland, who indicated that an increasing maternal age is a risk factor for G-DM [13,14]. It is possible that among women detected with gestational diabetes, some of them were already affected before pregnancy. Undiagnosed diabetes is prevalent, affecting primarily individuals living in low- and middle-income areas and among women at the age of childbearing, which varies from 1% at 18–24 years to 4% in those aged up to 44 years [15]. The higher weight among G-DM mothers may also be attributed to the additional weight gain associated with gestational diabetes itself. On the other hand, PG-DM mothers may have already had preexisting lifestyle modifications or medical interventions. The higher rank of gestations among affected mothers could be attributed to various factors, such as a higher prevalence of fertility treatments or an increased likelihood of having multiple pregnancies due to altered hormonal profiles associated with diabetes. In addition, the insulin resistant state achieved in late normal pregnancy is associated with subclinical inflammation, reduced adiponectin secretion and excess lipolysis among pregnant women diagnosed with G-DM [16]. In an experimental animal rat model, gestational diabetes with glucose dysregulation (G-DM) was induced using streptozotocin among pregnant mice. The effects of Tinospora cordifolia on insulin levels and resistance were examined. The study’s findings revealed that the combined effects of preserving beta pancreatic cells, enhancing insulin production, and reducing insulin resistance played pivotal roles in mitigating gestational complications associated with G-DM in pregnant mice [17]. 

We found that the cesarean section rate was higher, and the offspring were delivered earlier and were heavier among affected group compared to the healthier controls. In previous studies the authors also found a strong association between the non-elective cesarean section in the presence of G-DMM [18,19], while others found that controlled G-DM was associated with lower rates of cesarean delivery [20]. The higher rates of cesarean section in our cohort are comparable with previous research in the Omani population, which showed that 60% of G-DM women have undergone cesarean section and labor induction [21,22]. It is possible that the changing trends in the management of G-DM pregnancies could be shadowed by the rising of the cesarean rates in developing countries, similar to our studied population [23]. Diabetic pregnancies are often deemed higher risk due to potential complications, such as fetal macrosomia or increased difficulty in achieving vaginal delivery. Consequently, healthcare providers may opt for cesarean section as a precautionary measure to ensure the safety of both the mother and the infant.

In our study, we found a higher prevalence of respiratory disease, NICU admissions, and congenital heart defects among affected infants, which aligns with previous research in infants of diabetic mothers [24,25,26]. These complications may stem from an altered metabolic environment and potential hyperglycemia during pregnancy, which can affect fetal organ development and lead to adverse outcomes. The rate of respiratory disorders among IDM newborns was around 20%, which could be considered similar with previous a study [27], but lower compared to the study performed by Rosenn et al., which showed that G-DM is associated with prematurity and intrauterine growth restriction( stunted growth), which contribute mostly to the development of respiratory distress syndrome [28]. Nevertheless, it is plausible that the elevated weight and BMI of the affected mothers may have also played a role in the increased cesarean section rates, potentially contributing to the higher incidence of respiratory diseases in their offspring. However, it is worth noting that these factors alone may not fully explain the magnitude of the observed effect in the case group. The lower rate of intrauterine growth restricted (stunted growth) and the exclusion of preterm births would be responsible for the lower rates of respiratory disorder in our studied population. 

### Strengths and Limitation of the Study

A key finding in this study is that optimal blood glucose control during the last trimester of pregnancy did not reduce offspring morbidity. This suggests that achieving glycemic control solely in the final trimester may not ensure favorable perinatal outcomes for women diagnosed with gestational diabetes late in the second trimester, aligning with prior research on lipid transfer to the fetus [29,30] or on neurodevelopmental neonatal outcome [31]. A notable strength of our study lies in our rigorous selection criteria, which excluded mothers with confounding factors such as gestational hypertension, eclampsia, preeclampsia, intrauterine growth affected fetuses (stunted growth), and preterm delivery.

It is important to acknowledge several limitations of this present study. First, factors such as the severity and control of maternal diabetes, as well as potential confounding variables like maternal smoking, TORCH infections, or fertility treatments were not fully accounted for in the analysis. Moreover, our study lacks information on maternal lifestyle and caloric intake across different pregnancy phases, hindering an exploration of underlying mechanisms. Additionally, our study did not replicate the previously observed link between a family history of diabetes and gestational diabetes in the Omani population [32]. Another important limitation is the convenience sampling and relatively small size of the affected group compared to the previously mentioned research. Additionally, some patients were not aware of their family history of diabetes.

Our results suggest that even infants of optimally controlled diabetic mothers, both those with gestational diabetes (G-DM) and pre-gestational diabetes (PG-DM), have different characteristics and outcomes compared to infants of non-affected mothers. One possible mechanism is that gestational diabetes and maternal overweight is related to the abnormal oxidative stress, energy homeostasis, angiogenesis and insulin insensitivity, which are all tightly associated with negative consequences for both mother and her fetus [33,34]. The present study aligns with an earlier prospective cohort study, which has shown that an elevated incidence of neonatal morbidities can be attributed to factors beyond hyperglycemic control alone. These factors include progressive insulin resistance and inadequate insulin secretory capacity [35]. The study findings can be extended to other populations of IDMs with similar maternal anthropometric and medical traits. However, simply normalizing glycemic levels without addressing the broader factors, such as the gestational diabetes hormonal response, overweight status, dietary habits, lifestyle, and food environment, may not mitigate the risk of adverse perinatal outcomes. Future research should consider these factors, as well as data on euglycemic maternal status before pregnancy, to obtain a more comprehensive understanding of their influence on neonatal outcomes. 

## 5. Conclusions

This study highlights a distinctive aspect, as it reveals that adverse neonatal outcomes in infants of diabetic mothers persist despite optimal self-glycemic control, adding a unique dimension to existing research. These findings underscore the importance of preconception care, earlier detection and directed treatment of diabetes in women of childbearing age. Further studies exploring potential interventions and strategies to improve maternal and neonatal outcomes in this population are warranted.

## Figures and Tables

**Table 1:** Comparison of maternal characteristics of DM’s and controls.

Characteristics	Non-DM N = 138	IDMs N = 46	G-DM N = 38	PG-DM N = 8	*p* ^a^ Values		
					DM vs. Non-DM	G-DM vs. Non-DM	G-DM vs. PG-DM
Maternal age, Mean (SD)	28.9 (6.2)	32.1 (5.7)	31.6 (6.1)	34.7 (3.2)	0.002	0.02	0.04
Maternal weight, Mean (SD)	77.1 (13.9)	90.4 (13.6)	91.7 (14.2)	82.6 (6.1)	0.001	0.001	0.01
Maternal height Mean (SD)	163 (6.6)	164 (5.5)	163 (5.1)	167 (7.1)	0.4	0.7	0.2
BMI (Kg m^−2^) Mean (SD)	28.8 (4.9)	33.5 (5.3)	34.2 (5.3)	29.4 (2.8)	0.001	0.001	0.006
BMI > 30 N, %	30 (21.7)	34 (73.9)	30 (78.9)	4 (50)	0.001	0.001	0.01
BMI within [25–29.9] N, %	53 (38.4)	8 (17.4)	6 (15.7)	2 (25)	0.02	0.01	0.01
Family history of DM N, %	17 (12.3)	9 (19.5)	6 (15.7)	3 (37.5)	0.2	0.6	0.2
Family history of hypertension N, %	26 (18.8)	11 (23.9)	8 (21.1)	3 (37.5)	0.04	0.4	0.001
Family history of autoimmune disease N, %	12 (8.7)	8 (17.4)	8 (21.1)	0	0.01	0.02	-
Maternal smoking N, %	33 (23.9)	11 (23.9)	9 (23.7)	2 (25)	0.001	0.9	0.9
Gestations Mean, SD	1.4 (2.5)	2.2 (1.3)	1.4 (0.5)	2.3 (1.5)	0.2	0.6	0.06
Parity Mean, SD	1.1 (0.8)	1.7 (0.7)	1.7 (0.7)	1.3 (0.5)	0.7	0.9	0.2
GA at recruitment Mean, SD	28 (2.1)	28 (1.1)	28 (1.2)	28 (1.1)	0.8	0.7	0.6
Vaginal delivery N, %	64 (46.4)	14 (30.1)	14 (37)	0	0.01	0.02	0.04
Meconium strain amniotic fluid N, %	9 (6.5)	1 (2.2)	1 (2.6)	0	0.2	0.3	0.6
Cord blood pH Mean, (SD)	7.3 (0.1)	7.24 (0.1)	7.2 (0.1)	7.3 (0.1)	0.3	0.1	0.3
Oxygen use in delivery room (%)	5 (3.6)	5 (10.8)	2 (5.3)	3 (37.5)	0.05	0.6	0.007
Apgar’s score 1-min Median (range)	9.5 (0.7)	8.9 (0.9)	8.4 (1.4)	9.1 (0.7)	0.002	0.01	0.04
Apgar’s score 5-min Median (range)	9.8 (0.4)	9.6 (0.6)	9.2 (0.7)	9.7 (0.5)	0.02	0.08	0.03

^a^ Chi-square test or Fisher’s exact test, as appropriate: global test comparing distributions between cohorts. BMI body mass index, SD standard deviation, IDMs infants of diabetic mothers, PG pregestational diabetes. Data are expressed as mean or percentages.

**Table 2:** Anthropometric characteristics among included newborns.

Characteristics	Healthy N = 138	IDMs N = 46	G-DM N = 38	PG-DM N = 8	*p* ^a^ Values		
					DM vs. Healthy	G-DM vs. Healthy	G-DM vs. PG-DM
Gestational age weeks *Mean, (SD)*	39.7 (1.1)	38.1 (1.7)	38.2 (1.5)	37.7 (0.5)	0.1	0.1	0.01
Male *N, %*	62 (45)	22 (47.8)	(50)	(38)	0.6	0.6	0.5
Birth weight grams *Mean, (SD)*	3284 (394)	3413.1 (61)	3469.7 (571)	3143.7 (762)	0.02	0.02	0.2
Fenton percentile’s for birth weight, *Median (range)*	34 (97)	83 (94)	59 (31)	54.6 (35.3)	0.001	0.001	0.7
Length cm *Mean, (SD)*	52.5 (1.9)	53 (2.9)	53.4 (2.7)	51.1 (3.4)	0.02	0.05	0.04
Fenton percentiles for length, *Median (range)*	69 (93)	66 (98)	77 (23)	68.5 (25.6)	0.005	0.004	0.3
Cranial perimeter cm *Mean (SD)*	33.9 (1.5)	34 (1.6)	34.1 (1.5)	33.4 (1.9)	0.3	0.3	0.4
Fenton percentile for cranial perimeter *Median (range)*	27 (91)	45 (98)	41 (32)	38.8 (28.8)	0.05	0.05	0.8

^a^ Chi-square test or Fisher’s exact test, as appropriate: global test comparing distributions between cohorts. SD standard deviation, PG pre-gestational, G gestational, IDMs infants of diabetic mothers.

**Table 3:** Neonatal outcomes among cohorts.

Characteristics	Healthy N = 138	IDMs N = 46	G-DM N = 38	PG-DM N = 8	*p* ^a^ Values		
					DM vs. Healthy	G-DM vs. Healthy	G-DM vs. PG-DM
NICU admission %	6 (4.3)	11 (23.9)	6 (15.7)	5 (62.5)	0.01	0.06	0.04
Birth trauma %	7 (5.1)	6 (13)	4 (10.5)	2 (25)	0.1	0.2	0.4
Hypoglycemia, %	4 (3)	1 (2.2)	0	1 (12.5)	0.04	0.04	0.6
Respiratory disorder %	4 (3)	9 (19.5)	7 (18.4)	2 (25)	0.02	0.02	0.7
Polycythemia %	0	2 (4.3)	2 (5.3)	0	0.02	0.02	0.1
Phototherapy %	25 (18.1)	20 (43.5)	17 (44.7)	3 (37.5)	0.001	0.001	0.7
Congenital heart defects %	4 (3)	7 (15.2)	5 (13.1)	2 (25)	0.02	0.02	0.5
Myocardial hypertrophy %	6 (4.3)	12 (26)	9 (23.6)	3 (37.5)	0.01	0.001	0.5
Total congenital defects %	4 (3)	5 (10.9)	4 (10.5)	1 (12.5)	0.03	0.03	0.8
Days of hospital stay. Mean (SD)	3.5 (1.3)	4.7 (2.7)	4.2 (1.8)	7.4 (4.7)	0.01	0.01	0.02

^a^ Chi-square test or Fisher’s exact test, as appropriate: global test comparing distributions between cohorts. NICU = Neonatal intensive care unit, SD = standard deviation, PG pregestational, IDMs infants of diabetic mothers, Numbers are N (%) unless otherwise stated.

**Table 4:** Odds and 95% CI of neonatal outcomes by groups.

	Respiratory Disorder OR 95% CI	Congenital Heart Defects OR 95% CI	NICU Admission OR 95% CI
PG-DM	4.5 (0.8–25.1)	5.6 (0.9–31.7)	22.5 (4.7–107.1)
G-DM	4.7 (1.5–14.8)	3.3 (0.9–10.9)	2.3 (0.7–6.6)
IDMs	7.2 (2.1–24.6)	5.3 (1.5–19.1)	7.3 (2.4–22.9)
Infants of unaffected mothers ^#^	Reference	Reference	Reference

PG pregestational, IDMs infants of diabetic mothers, CI confidence interval, OR odds ratio. ^#^ infants of unaffected mothers are the reference group.

## Data Availability

The data that support the findings of this study are available from the corresponding author L.M.S. upon reasonable request.

## Supplementary Materials

The following supporting information can be downloaded at: https://www.mdpi.com/article/10.3390/medicina59101768/s1, Title: Lifestyle guidance: diet and physical activity during third trimester of pregnancy [36,37,38,39,40,41,42,43,44,45].

## Abbreviations

BMI = Body mass index, CI = Confidence interval, GA = Gestational age, G-DM = Gestational diabetes mellitus, ICD = International classifications of disease, IDM = Infants of diabetic mothers, NICU = Neonatal intensive unit, OC = Optimally controlled, OR = Odds ratio, PG-DM = Pregestational diabetes mellitus, SD = standard deviation, SPSS = Statistical package for social science, T1DM = Type 1 diabetes mellitus, T2DM = Type 2 diabetes mellitus, TORCH = Toxoplasmosis, rubella, cytomegalovirus, herpes simplex.
