# Supplementary material for: Optimally Controlled Diabetes and Its Influence on Neonatal Outcomes at a Level II Center: A Study on Infants Born to Diabetic Mothers
Source: Medicina (Kaunas). 2023 Oct 4;59(10):1768. doi: 10.3390/medicina59101768 (PMC10607977; doi:10.3390/medicina59101768)
Supplement: Supplementary file 1 [file medicina-59-01768-s001.zip › medicina-2618920-supplementary.pdf]

Lifestyle guidance: diet and physical activity during third trimester of pregnancy

1. Three balanced meals throughout the day with three to four snacks per day [1]
2. Total energy intake to approximately 2000 kcal per day [2]
3. Total carbohydrate intake to 40-55% of the total energy intake [2]
4. No intakes of sugar sweetened beverages, high intake of fruits and vegetables [3]
5. Total fat intake to 30% of total energy, and substitute monosaturated fatty acids for saturated and trans-fatty acids [4]
6. Protein intake to 20-30% of total energy [5, 6]
7. Vitamin D supplement [7]
8. Probiotic supplements [8]
9. Walking for 30 min per day [9, 10]

References

1. Most, J.; Dervis, S.; Haman, F.; Adamo, K.B.; Redman, L.M. "Energy intake requirements in pregnancy." Nutrients 11.8 (2019): 1812. doi.org/10.3390/nu11081812
2. Institute of Medicine. Dietary Reference Intakes for Energy, Carbohydrate, Fiber, Fat, Fatty Acids, Cholesterol, Protein and Amino Acids. Washington, DC: National Academies Press; 2005
3. Meyers, L.D.; Hellwig J.P.; Otten, J.J. eds. Dietary reference intakes: the essential guide to nutrient requirements. National Academies Press, 2006.
4. Villegas, R.; Salim, A.; Collins, M.; Flynn, A.; Perry, I. (2004) Dietary patterns in middle-aged Irish men and women defined by cluster analysis. Public Health Nutr 7(8), 1017–1024. doi:10.1079/PHN2004638
5. Moore, V.M.; Davies M.J.; Willson, K.J.; Wosley, A.; Jeffrey, R.S. Dietary composition of pregnant women is related to size of the baby at birth. J Nutr. 2004; 134:1820–1826. doi.org/10.1093/jn/134.7.1820
6. Mousa, A.; Naqash, A.; Lim, S. Macronutrient and micronutrient intake during pregnancy: an overview of recent evidence. Nutrients 11.2 (2019): 443. doi.org/10.3390/nu11020443
7. De-Regil, L.M.; Palacios, C.; Pena-Rosa J.P. Vitamin D supplementation for women during pregnancy." Cochrane database of systematic reviews 1 (2016). DOI: 10.1002/14651858.CD008873.pub3.
8. Baldassarre, M. E; Palladino, V.; Amuroso, A.; Pindinelli, S.; Mastromarino, P.; Fanelli, M.; Di Mauro, A.; Laforgia, N. Rationale of probiotic supplementation during pregnancy and neonatal period. Nutrients 10.11 (2018): 1693. doi.org/10.3390/nu10111693
9. Committee on Obstetric Practice (2002) ACOG committee opinion. Exercise during pregnancy and the postpartum period. Number 267, January 2002. American College of Obstetricians and Gynecologists. Int J Gynecol Obstet 77, 79–81.
10. Royal College of Obstetricians and Gynecologists (2006) Exercise in Pregnancy (RCOG Statement 4). http://www.rcog.org.uk/womens-health/clinicalguidance/exercise-pregnancy (accessed June 30, 2023)
